# INFIX/EXFIX: Massive Open Pelvic Injuries and Review of the Literature

**DOI:** 10.1155/2016/9468285

**Published:** 2016-07-14

**Authors:** Rahul Vaidya, Kerellos Nasr, Enrique Feria-Arias, Rebecca Fisher, Marvin Kajy, Lawrence N. Diebel

**Affiliations:** ^1^Detroit Medical Center, 4D University Health Center, Detroit Receiving Hospital, 4201 Street Antoine Boulevard, Detroit, MI 48201, USA; ^2^4D University Health Center, Detroit Receiving Hospital, Wayne State University, 4201 Street Antoine Boulevard, Detroit, MI 48201, USA

## Abstract

*Introduction*. Open pelvic fractures make up 2–5% of all pelvic ring injuries. Their mortality has been reported to be as high as 50%. During Operation Enduring Freedom protocols for massive open pelvic injuries lead to the survival of injuries once thought to be fatal. The INFIX is a subcutaneous anterior fixator for pelvic stabilization which is stronger than external fixation. The purpose of this paper is to describe the use of INFIX and modern algorithms for massive open pelvic injuries.* Methods*. An IRB approved retrospective review describes 4 cases in civilian practice with massive open pelvic injuries. We also review the modern literature on open pelvic injures.* Discussion*. Key components in the care of massive open pelvic injuries include hemorrhage control by clamping of the aorta or REBOA when necessary and fecal/urinary diversion. The INFIX can be used internally, as a partial INFIX partial EXFIX, or as an EXFIX. Its low profile allows for easy application of wound vacs and wound care and when subcutaneous avoids pin tract infections.* Conclusion*. Massive open pelvic injuries are a difficult problem. Following modern protocols can help prevent mortality.

## 1. Introduction

Open pelvic fractures make up 2–5% [1–6] of all pelvic ring injuries and their mortality has been reported to be as high as 50% [1, 2, 7–9].

One of the most complex open pelvic fracture injury patterns is the high-energy improvised explosive device blast suffered by a dismounted soldier presenting with traumatic bilateral lower extremity amputations including pelvic and perineal involvement. During Operation Enduring Freedom protocols for the care of these individuals lead to survival of injuries once thought to be fatal [10]. These lessons have advanced the care of massive open pelvic fractures and extremity injuries and can be applied to civilian injuries as well.

The INFIX is an Anterior Subcutaneous Pelvic Fixation Device which is biomechanically stronger than an external fixator due to its internal profile, has the advantage of improved patient comfort/mobility, eliminates pin tract infections, and can serve as temporary and then definitive fixation once the posterior pelvis is stabilized [11–17]. We think this is the ideal tool for stabilization out of war and disaster zones where temporary stabilization often remains the method of treatment for several weeks or as definitive treatment. It can be used as an INFIX, as a partial INFIX partial EXFIX (INFIX/EXFIX), and as an EXFIX as it is biomechanically stronger than a 2-pin supra-acetabular EXFIX due to its low profile [15, 18].

The purpose of this paper is to describe (1) the use of pelvic INFIX in complex open pelvic injuries; (2) the use of a complex open pelvic fracture protocol developed in OEF in civilian practice; and (3) review the literature for open pelvic injuries.

## 2. Methods

An IRB approved retrospective study was performed on 4 open pelvic fractures that had significant soft tissue injury. All were treated by the ATLS protocol, pelvic INFIX device, and laparotomy with control of the vessels to the lower limbs when indicated. Two case examples are given. We also reviewed the available literature on open pelvic fractures and with regard to acute care; initial surgical procedure; hemorrhage control; soft tissue care; bowel injury; urogenital injuries; definitive pelvic stabilization; and the INFIX device.

## 3. Case Examples

### 3.1. Case  1

**Figure 1:**
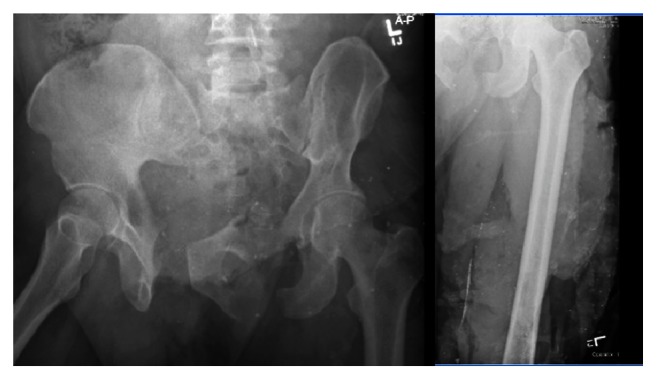
(Case  1) 41 yo male crushed by fork lift. APC 3 massive open pelvic ring injury and mangled left lower extremity arrived in extremis. The left leg with a vascular injury was debrided, had an above knee amputation, and eventually resulted in a hip disarticulation.

**Figure 2:**
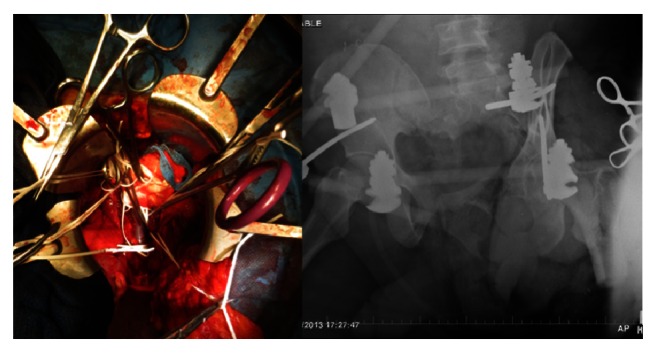
Laparotomy was performed where intra-abdominal bleeding was controlled and then the aorta and IVC clamped low in the abdomen to stop the blood loss from his pelvis and left lower extremity.

A 41-year-old healthy male was crushed by a forklift and brought to our ER in hemorrhagic shock. He suffered a massive open injury to his left leg and pelvis (Figure 1). ATLS protocol was followed, the massive transfusion protocol was activated, and a pelvic binder applied for his APC 3 pelvic injury. He was transferred to the OR for intra-abdominal bleeding identified on FAST exam and a lack of response to transfusion. A laparotomy was performed where intra-abdominal bleeding was eventually controlled and then the aorta and IVC were clamped low in the abdomen to stop the bleeding from his pelvis and left lower extremity. During this time the left leg wound was debrided and irrigated, and it was realized that a high above knee amputation was required as the leg was nonviable. A pelvic EXFIX was placed, and the patient was given a high diverting colostomy. Urology placed a suprapubic catheter and removed an avulsed devascularized left testicle. Once the vessels were ligated distally, the aorta and vena cava were released (Figure 2). He was transferred to the ICU. He received 31 units of PRBC, 18 units of FFP, 25 units of platelets, and 5 units of cryoprecipitate over the first 24 hrs and stabilized hemodynamically.

**Figure 3:**
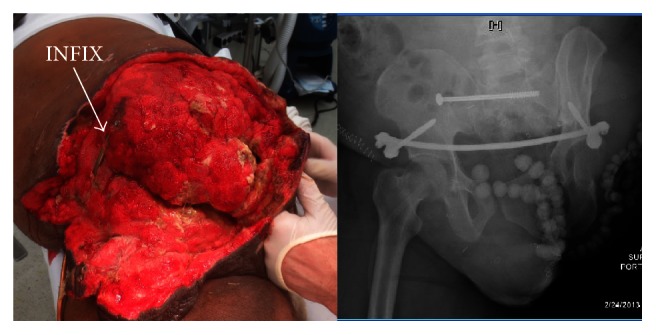
INFIX/EXFIX, as well as a percutaneous SI joint screw to stabilize the pelvis then antibiotic beads and wound vacs to combat the soft tissue injury.

The patient underwent serial debridement because of ongoing and progressive tissue necrosis in the stump, treated with negative pressure wound vacs and antibiotic beads. The above-the-knee amputation was converted to a hip disarticulation but the patient still had a massive wound over his pelvis and his lower abdomen. The wound vacs were difficult to apply with the external fixator and the pelvis was still relatively unstable so the external fixator was removed and reduction of his pelvic ring injury was performed with a subcutaneous anterior internal fixator, as well as one percutaneous SI joint screw (Figure 3). This presented somewhat a challenge given the extent of the patient's open wounds on the left hemipelvis leaving part of the internal fixator exposed to air on the left side. The construct stabilized the injury and the low profile of the INFIX allowed easy application of the wound vac.

**Figure 4:**
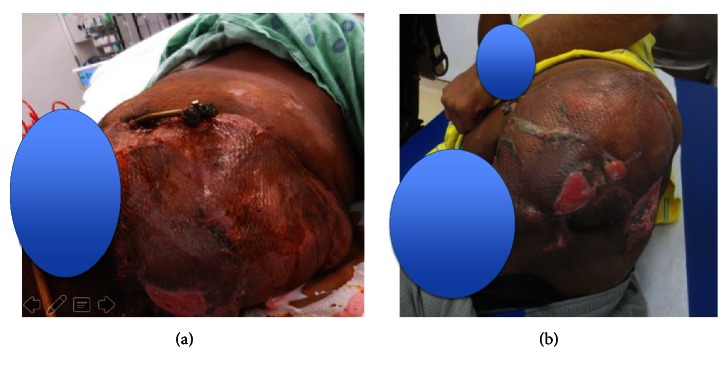
(a) Wound healing and (b) the fixator removed.

The wound and patient stabilized, and skin grafts were applied to the defect once good granulation tissue was apparent around the pelvic INFIX (Figure 4).

The patient underwent removal of his suprapubic catheter and eventual reanastomosis of the bowel once all the wounds had healed. He was fortunate to have functional return of his urethra.

The traumatized area under the skin grafts has had formation of heterotopic bone which required removal on 2 occasions due to pain with prosthetic fitting. This is finally underway 2 years after the injury.

### 3.2. Case  2

**Figure 5:**
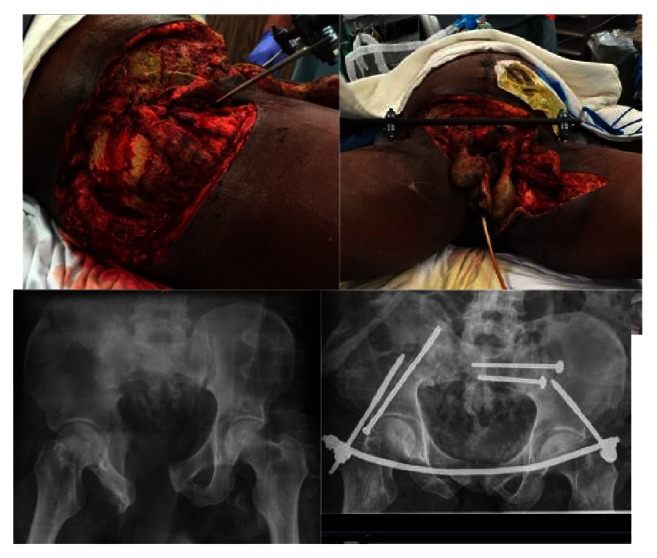
This is a 62-year-old male who was involved in a motor vehicle accident. His open injury was missed initially when he arrived in extremis and developed a massive pelvic soft tissue infection requiring debridement over the first 3 days.

**Figure 6:**
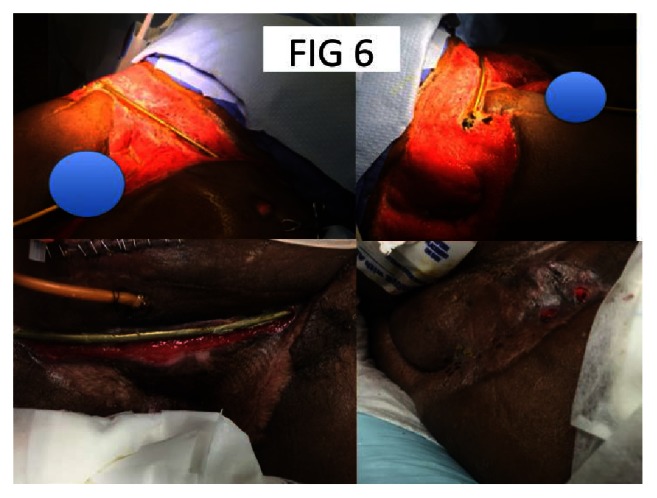
After fixation bowel and urinary diversion and wound management.

This is a 62-year-old male who was involved in a motor vehicle accident. His open injury was missed initially when he arrived in extremis and developed a massive pelvic soft tissue infection requiring debridement over the first 3 days. He had a colostomy, superpubic catheter, percutaneous reduction of his iliac wing, SI screws, and an anterior INFIX. His testicles were implanted under the medial thigh and he had extensive wound care (Figures 5 and 6). This was followed by a long ICU course and dressing changes daily. The wound granulated in and the INFIX was removed once we felt that there was adequate healing (5 months). The patient had skin grafts and still has a colostomy.

### 3.3. Case  3

A 23 yo male was involved in an MVA where his pelvis was impaled by a pole. ATLS protocol was initiated and he was taken emergently to the OR to stabilize his bleeding. The aorta was clamped, the pelvis packed and antishock SI screws with a EXFIX using low profile INFIX implants were applied, and a colostomy was performed. The aorta was released and right iliac vessel ligated but bleeding persisted despite packing and angiography. The patient was given a massive transfusion with 30 PRBC's 25 units platelets and 30 u plasma but expired on POD #3 in the ICU due to head injury and ARDS.

### 3.4. Case  4

A 21 yo male was involved in a motorcycle accident where he hit his perineum hard on the motorcycle gas tank and suffered an APC 3 injury with herniation of his bowels. He was hemodynamically stable after ATLS protocol with resuscitation. He went to the OR for an I and D, a colostomy, suprapubic urinary catheter, and stabilization of his pelvis with an INFIX and SI screws. He did well postoperatively and at latest follow-up had his colostomy reconnected and urethra reconstructed (see Table 1).

## 4. Discussion

Pelvic fractures are a major cause of mortality among trauma patients. These fractures often result from a high-energy trauma and cause injury to other parts of the body. Associated injuries are therefore common and may affect outcomes in patients with a pelvic fracture. Mortality in patients sustaining pelvic fractures has been reported to be 4–15% [19–24]. Early deaths are attributed to hemorrhage or injuries of the central nervous system, and delayed deaths are reportedly due to sepsis and multiple organ failure [8, 25–29]. Various risk factors for mortality have been observed including increasing age, shock on initial presentation (as defined by a systolic blood pressure <90 mmHg) [19, 20, 23, 25, 27, 30, 31], severity of associated injuries [20, 24, 32, 33], and open pelvic fractures. Open pelvic fractures make up 2–5% [1–6] of all pelvic ring injuries and their mortality rate has been reported to be as high as 50% [1, 2, 7–9]. Mortality is usually related to the same causes as closed pelvic fractures. Over the last 30 years, the mortality rate from pelvic fractures has decreased due to the implementation of multidisciplinary protocols, improved hemorrhage management [27], identification of open injuries, advances in critical care medicine, aggressive fracture management, and early fecal/urinary diversion for open pelvic injuries.

## 5. The Initial Surgical Procedure Open Pelvic Fracture

Hemorrhage control, surgical debridement, and pelvic volume reduction plus stabilization are the priorities of the index operation. Secondary priorities are stabilization of long bone fractures through external fixation, bladder repair, potential colonic diversion, and irrigation and debridement of open injuries. A multidisciplinary team approach using general, orthopaedic, and urologic surgeons working simultaneously is the most effective method for care of these unstable patients. Emergency pelvic stabilization can be accomplished with a pelvic binder in the trauma bay, which should be converted intraoperatively to an anterior external frame or c-clamp in centers that utilize this device. The patients require massive transfusions with packed red blood cells, plasma, and platelets in what has deemed to be the optimum ratio of 1 : 1 : 1.

## 6. Hemorrhage Control

Hemorrhage control is accomplished by laparotomy where intra-abdominal bleeding is present. When bleeding is not controlled with these maneuvers, the addition of retroperitoneal pelvic packing (if this expertise is available) is indicated [34, 35]. With communication to the outside (open injury), the bleeding often continues as there is no contained compartment. At this point a decision needs to be made whether or not to clamp or ligate the internal iliac artery, clamp the aorta [10], or use Resuscitative Endovascular Balloon Occlusion of the Aorta (REBOA) [36]. In patients with severe open pelvic or abdominal injuries, temporizing proximal vascular control of the iliac vessels or aorta via a celiotomy or the retroperitoneal approach is an option. The final level of vascular control is a balance of preventing exsanguination and still advancing temporary vascular control to the most distal and viable level. This is critically important in cases requiring hip disarticulation or hemipelvectomy [10], where it is difficult to control bleeding more distally as the vessels often retract into the pelvis.

Angiography and embolization have been shown to be highly effective but should be reserved for those patients who continue to bleed after laparotomy and the index surgical procedure. In institutions where pelvic packing expertise is not yet available, it is the next step after laparotomy and temporary pelvic fixation. If laparotomy is not indicated, angiography is the next step after reduction of pelvic volume with a binder, external fixation, or c-clamp. Angiography may be time consuming and is only useful for arterial bleeding [37].

Once bleeding control is achieved other surgical procedures can proceed.

## 7. Soft Tissue Care

Adequate index surgical debridement is a critical step in preventing later risks of infection. Systematic sharp debridement of all foreign material, nonviable skin, subcutaneous tissue, fascia, periosteum, and bone is performed back to viable, healthy tissue. Subsequently a meticulous washout, possibly utilizing the pulsed lavage technique, is necessary [10, 37–39]. This is followed either by open wound treatment or vacuum sealed dressings, which allow adequate drainage of the wound. It is important to note that with large fresh wounds the use of a wound vac can lead to excessive hemorrhage. We often refrain from using these devices until the second I and D procedure and once bleeding has stopped. One can expect to be involved in multiple debridement as tissue ischemia continues to evolve over several days. Daily or every second day serial debridement may be necessary [10].

In massive injuries, where amputation of a leg is necessary, one can harvest skin from the amputated extremity for later use. Healthy flaps of skin, subcutaneous tissue, and muscle are kept as attached flaps for later coverage. Wounds can be closed at a later time by delayed primary closure. If the wounds are clean, they can be closed by secondary intention and covered with STSG or rotational myocutaneous flaps.

## 8. Bowel Injury

Early diverting colostomy or ileostomy and distal rectal washout of residual faeces has been the widely accepted treatment for all open pelvic fractures [37]. It has significantly reduced infective complications and consequently reduced mortality [4, 38–41]. Fecal diversion is preferably performed as a loop but can also be performed as an end colostomy [4, 38–41]. Potential sites of subsequent orthopaedic incisions, external fixator pins, and suprapubic cystostomy should be borne in mind when locating the stoma [1, 42]. Open perineal wounds involving the rectum require fecal diversion with early sphincter repair and local open wound management [43, 44].

## 9. Urogenital Injuries

If there is any evidence of urethral injury or a positive cystourethrogram, a suprapubic Foley catheter needs to be inserted during the index procedure to ensure the diversion of urine. This is to prevent sepsis from infected urine and to monitor flow for resuscitation. Realignment of the urethra can be accomplished if the patient is stable and the expertise is available; otherwise once diversion is accomplished, realignment can wait [45]. This is accomplished with fluoroscopy or urethroscopy, and both antegrade imaging and retrograde imaging are required to realign the urethra prior to passing a Foley catheter. Reduction of the anterior pelvic ring is helpful for urethral realignment [37].

Intraperitoneal bladder ruptures should be repaired early while extraperitoneal bladder ruptures can be diverted or repaired. In complicated extraperitoneal bladder ruptures (if the patient is stable and the expertise is available) immediate repair may be important especially if an anterior fracture has to be instrumented. These are recommendations by the American Urological Society based on the available evidence [45].

In vaginal lacerations, early definitive repair of the lesion with absorbable sutures is recommended in order to prevent abscess formations. Care must be taken to close the tear while not injuring the uterine arteries, which lie along the lateral borders of the vaginal vault [37, 38].

Scrotal and perineal lacerations should be debrided and irrigated. Like other soft tissue injuries, they can be closed if the wound beds are good. Evaluation of testicular viability should be performed by the urologic or general surgeon [45]. When there is lack of soft tissue coverage, they can be implanted under viable areas like the inner thigh until coverage is possible.

## 10. Definitive Pelvic Stabilization

Eventually the pelvic ring has to be reconstructed in a patient who is now stable and has survived the initial trauma using damage control orthopaedics. The principles of fixation are the same as for closed fractures except for the soft tissue injury [37, 39, 44]. Posterior fixation can be placed percutaneously after reduction using sacroiliac screws and lumbopelvic fixation for comminuted posterior injuries. Iliac wing fractures can be fixed with plates and screws if soft tissue allows, as external and percutaneous options are not as effective. The anterior pelvic ring, where most soft tissue injuries occur, can be stabilized with definitive external fixation [37, 39, 44] or an anterior internal fixator which we have used as an INFIX/EXFIX. In massive injuries this facilitates the use of vacuum assisted closure techniques as the INFIX can be completely covered with this [11].

The pelvic INFIX was developed for indications where anterior external fixation is commonly used [11]. We feel it is a good replacement for any anterior fixation where one might otherwise use a plate, an EXFIX, or ramus screws [11–18]. It is made with custom pedicle screws (long lengths 80–120 mm) of 7 or 8 mm diameter and sits under the skin in an area we call the “Bikini Line” [13]. It is more stiff than an anterior external fixator [15, 18] that allows patients increased mobility and is particularly useful in obese patients [17]. We have found it particularly useful in conjunction with a laparotomy, in combination with internal fixation as an acetabular fracture, and feel it could be important in patient transportation with pelvic injuries as external fixation pins get infected when left in place for 2 weeks or longer. The INFIX can be loosened allowing definitive reduction and fixation for the posterior pelvic injury and then reused to stabilize the anterior pelvis definitively. We have used this device as an internal fixator, INFIX/EXFIX, partially covered and partially exposed as we have demonstrated in this paper, and as an external fixator when there is no soft tissue coverage. Its low profile makes it much stiffer than an external fixator and it is easy to cover under a wound vac when needed. Originally we did not use INFIX during damage control as the parts were not readily available so we converted external fixators to INFIXs when necessary. However as sets become available or as in our case we have an INFIX set in our OR, we often will do an INFIX as the index and definitive procedure. We recommend the use of INFIX with the appropriate posterior fixation and have only used it as a standalone device during transport or on certain APC2 injuries with symphyseal disruption.

The complications of INFIX have been previously described and include heterotopic ossification which is usually asymptomatic and implant loosening or failure which is rare and often related to technique [17]. Lateral femoral nerve irritation is common but often goes away once the implant is removed [17]. There have been reports of femoral nerve palsy with the INFIX procedure [46]. We have not seen it in our series and feel it is related to sinking the screws too far into the bone. The ideal scenario is to leave the rod very superficial and fix the pedicle screw height so that the rod sits right at pedicle screw head level. The technique is freely available as a video at the OTA video gallery online [47].

## 11. Conclusion

Hemorrhage control in massive open pelvic fractures can be difficult because of communication to the outside. Bleeding may continue after pelvic volume is reduced and the pelvis stabilized as there is no contained compartment. Clamping of the aorta or REBOA may be necessary. Early recognition of the open injury with fecal/urinary diversion has resulted in increased survival. The INFIX is a tool to stabilize the pelvis which is stronger than anterior external fixation. Its low profile allows for easy application of wound vacs for wound care, can hold the pelvic stable, and when placed internally for transport avoids infected pin tracks.

## Figures and Tables

**Table 1:** Case summaries.

Case	Injury	General surgery	Urology	Orthopaedics	Outcome
1 41 yo forklift- crush ISS 20	APC 3 massive open injury Crushed pelvis Extremis	Pelvic packing, temporary Aortic control clamp, colostomy	Suprapubic, orchiectomy	INFIX/EXFIX posterior SI screw, hip disarticulation	Survived to prosthetic fitting, bowel hooked up, Complication with heterotopic bone prosthetic fitting

2 62 yo MVA ISS 18	LC3 open Massive anterior open injury Extremis	Colostomy, debridement, implanted testicles in thigh	Suprapubic	INFIX/EXFIX, iliac screw posterior SI screws	Wound healed, pelvis healed, skin healing

3 21 yo MVA pelvis impaled by a pole ISS 45	APC3 open Massive anterior and posterior wounds, extremis	Colostomy, aortic control clamp packing	Suprapubic	INFIX antishock SI screws	Patient died in ICU POD #3 Rt tibia open fx, Rib fx pneumothorax bilateral pulmonary contusion

4 21 yo MVA motorcycle ISS 18	APC3 massive perineal wound with bowel hernia	Colostomy laparotomy	Suprapubic catheter	INFIX SI screws	Survived hooked up doing fine
